# Recognising Basic Health Literacy Capabilities: An Explorative Study on the Relevance of Health-Related Information in the Support of People with Profound Intellectual and Multiple Disabilities

**DOI:** 10.3390/ijerph192416874

**Published:** 2022-12-15

**Authors:** Timo Dins, Caren Keeley

**Affiliations:** Chair for Pedagogics and Rehabilitation for Persons with Intellectual and Profound Disabilities, Department of Rehabilitation and Special Education, Faculty of Human Sciences, University of Cologne, 50931 Cologne, Germany

**Keywords:** health literacy, people with intellectual disability, people with profound intellectual and multiple disabilities, participatory research, qualitative research, responsiveness

## Abstract

*Background*: People with profound intellectual disabilities represent a vulnerable and heterogeneous population whose health-related needs and questions often remain unheard. One reason for this is that they are usually unable to participate in verbal communication. However, there is also a lack of suitable approaches to communicate health-related information to them according to their capabilities. The research presented in this paper addresses this gap. *Methods:* Following grounded theory methodology, we used a multimethod approach. Based on a theoretical analysis, a Delphi study (*n* = 14) was conducted as a starting point to map the research field. In a second step, these findings were incorporated into an online survey targeting disability care professionals (*n* = 111). Three field studies supplemented the data, encompassing a variety of ethnographic methods. *Results*: People with PIMD have basic health-related capabilities that can improve their health literacy. Nevertheless, their support environments have to take over many health literacy-related requirements by proxy or substitution. One of the most important tasks is to engage health information in an individualised way. *Conclusions*: The findings underline the importance of focusing on more basic capabilities and intersubjective approaches in health literacy research and practice, especially regarding new perspectives on the inclusion of previously marginalised populations (such as people with PIMD).

## 1. Introduction

People with profound intellectual and multiple disabilities (PIMD) are considered to be particularly vulnerable, especially regarding their physical and mental health [1]. However, since they typically do not communicate verbally, their health-related needs, demands and questions often remain unheard—also in health literacy research. This article aims to address this desideratum by presenting the approach and main findings of the research project “Communicating (in) the Crisis” (ComCri), which focuses on the vulnerabilities and the health literacy needs of people with PIMD and their supporters. The project explored the following objectives:

1. Exploring the vulnerabilities and communicational needs of persons with PIMD in the context of the COVID-19 pandemic and beyond.
2. Exploring the relevance and accessibility of health-related information for persons with PIMD and their supporters in the context of the COVID-19 pandemic and beyond.
3. Deriving recommendations for action for good practice in communicating health-related information

This article outlines the multimethod approach we designed to achieve these objectives. By focussing on the second objective, our key results on the relevance and accessibility of health-related information in social contexts are described and discussed. Before presenting the methodology and results, it is necessary to describe the target group of this paper (and their health-related needs) in more detail (1.1) and to briefly address the question of how this group of people is considered in health literacy concepts and research (1.2).

### 1.1. People with Profound Intellectual and Multiple Disabilities

People with PIMD represent a very heterogeneous population. Most descriptions of this group of persons refer to medical classifications [2,3,4]. Under Code 6A00.3 (“Profound Disorder of intellectual development”), the latest and 11th revision of the International Statistical Classification of Diseases and Related Health Problems (ICD-11) include the following key characteristics: “A profound disorder of intellectual development is a condition (…) characterised by significantly below average intellectual functioning and adaptive behaviour that are approximately four or more standard deviations below the mean. (…) Affected persons possess very limited communication abilities and capacity for acquisition of academic skills is restricted to basic concrete skills. They may also have co-occurring motor and sensory impairments and typically require daily support in a supervised environment for adequate care.” [5]

In addition, health impairments are often compounded: “Studies, mainly from high income countries in Europe, North America, and Australia, indicate that in comparison to the general population people with ID are more likely to suffer from poor physical (…) and mental health (…), and that their life expectancy tends to be lower (…).” [1] What is described here for people with intellectual disability (ID) applies equally to people with PIMD. It can be assumed that these potential health challenges are much more profound, because people in this group are confronted with multiple medical conditions due to frequently occurring neurological and physical-motor impairments as well as other impairments that are usually syndrome-specific. Accordingly, health problems such as epilepsy, chronic respiratory diseases, nutritional or sleep problems [2] are often interrelated (e.g., when food intake is difficult due to chronic respiratory disease).

Based on the described impairments, it becomes clear how important (social) support is for this group of people and that lifelong dependence can therefore be regarded as a living condition that shapes everyday life experiences and individual biographies.

It can be noted that the majority of people with PIMD communicate non-verbally and use body language to express themselves and their needs [6]. “Due to the fact that these signals are highly individual, the number of those interaction partners who are actually capable of understanding and appropriately reacting to these signals is very restricted” [7]. This also applies to the communication of and about health-related information, which is of particular relevance for this group of people.

Another challenge is the often-limited possibilities to engage and interact with the surrounding world. This implies access to any educational content, information, the environment, etc., so supporting persons must always and individually consider the questions: How does the person interact with the world? Which learning opportunities does he or she prefer to use? Based on cultural-historical activity theory [8], it can be stated that a person always actively engages with the world—regardless of his or her individual capabilities. According to activity theory, a person does not only learn through reasoning or conceptual thinking. Learning evolves not only in the early stages of development but also from active (e.g., manipulative) interaction with everyday objects or from processes of perception-based engagement with the objects and subjects of the world. Of particular importance are interactions with familiar and close caregivers, particularly with shared attention to common objects. People with PIMD learn best when they have these opportunities for perception-based, manipulative and collaborative or joint engagement with the world [9,10].

These needs are hardly given any consideration. This became particularly apparent during the COVID-19 pandemic when there were barely any concepts or ideas for the target-group-specific communication of these measures of infection protection [11]. This resulted in a high degree of uncertainty both for people with PIMD and for their support environments. It also underlines the responsibility the support persons of people with PIMD have with regard to these demands. “However, nurses or caregivers are usually not trained to provide understandable health-related information in a corresponding manner. These aspects indicate that people with intellectual disabilities are a vulnerable group in terms of health, health literacy, health care, and everyday life support. Therefore, it is urgently necessary to include these people in the discussion about health literacy.” [12]

### 1.2. Health Literacy and Persons with (Profound) Intellectual Disabilities

Due to their high health-related vulnerabilities, people with PIMD need comprehensive support in all aspects of health care [1,12]. An integral part of their health-related support involves engaging with health-related information. In order to increase accessibility and reduce exclusion, it is necessary to enable people with PIMD and their supporters in this field, i.e., to recognise and enhance their (basic) health literacy capabilities. Based on Sørensen et al. [13], health literacy is commonly understood as “people’s knowledge, motivation and competences to access, understand, appraise, and apply health information in order to make judgements and decisions in everyday life concerning healthcare, disease prevention and health promotion to maintain or improve quality of life during the life course”.

However, it is now often emphasised that health literacy conceptualisations that focus on individual abilities and possibilities fall short. Rather, health literacy should be understood as a relational concept, thus, as the interaction of individual skills and competencies (personal or individual health literacy) and environmental and systemic factors [14]. Here, starting points for the health literacy of people with PIMD who are highly dependent on their environments can be identified. For this group of people in particular, interactive or communicative health literacy concepts appear to be particularly fruitful [15]. “Nevertheless, due to the particularities of people with intellectual disabilities described above, we question whether the application of common health literacy understanding to people with intellectual disabilities without further consideration or testing is feasible. Moreover, there is a lack of scientific evidence for a target-group-specific conception of health literacy within this group.” [12] Our research takes into account the models described above and makes an attempt to expand existing approaches to incorporate the perspective of people with PIMD.

One challenge we had to face in our research is the fact that there is still “a lack of insights into key aspects of accessing and using information that takes into account the particularities of people with intellectual disabilities” [12]. According to Latteck & Bruland, people with ID (and even more with PIMD), therefore, can be considered a “hidden population” [16]. In their review of 12 studies dealing with the health literacy of people with ID, the authors show that many health literacy interventions and studies presuppose skills and capabilities that this group of people often does not have. Consequently, this may also affect their consideration in health literacy research.

However, there is now a number of studies [17,18,19] that suggest that people with intellectual disabilities can develop an understanding of health issues if they receive the appropriate support. Yet, people with PIMD still remain largely invisible in these research activities. This may be due to the fact that these people can often only be reached with individualised approaches (see Section 1.1 of this article). In our research, we followed the assumption that, in this respect, it is crucial to take a closer look at more basic skills. In concrete terms, this means including those basic individual health-related capabilities that are also available to non-literate or non-numerate persons [20] and exploring the role of their support environments in facilitating understanding.

### 1.3. Aims and Research Questions

It can be stated that at the present time, there is still little or no knowledge about (how to foster and expand) the health literacy of people with PIMD. It should already have become clear that dealing with health-related information is of great importance in supporting people with PIMD. This article addresses these requirements by, first, presenting the multimethod research approach of a qualitative study, which makes a significant contribution to meeting this desideratum. On the other hand, our paper presents the results that were gained with this approach, focusing on how to engage with health-related information for and by people with PIMD. Therefore, we address the following questions:

- How do people with PIMD engage with health-related information?
- How do their care professionals engage with health-related information that is relevant for people with PIMD?
- How can their care professionals communicate health-related information to people with PIMD?

Before presenting and discussing our findings to these questions, we will now briefly outline the comprehensive multimethodological design of the project.

## 2. Materials and Methods

The presented research follows a qualitative approach, which is based on hermeneutics [21] as well as on (ethnographic) field research. In order to meet both basic research and applied research interests, the approach is explorative and accordingly triangulated. For this purpose, a multimethod and circular approach was used that derives benefit from iterative additions to data collection, analysis and interpretation. This process is also evident in the framework of the overall analysis, which is based on elements of grounded theory methodology (GTM) according to Strauss & Corbin [22], as can be seen in the corresponding passages (see Section 2.4 of this article).

The research process started with a review of the current state of research on the thematic focuses of the project (see the Introduction). The review aimed to identify and analyse existing studies and relevant research in this field. As a structural tool, the work was based on the Wiki principle [23], and a corresponding online tool was used, which made it possible to write in a collaborative way, in which existing content can be changed and supplemented and linked and expanded among each other. The findings helped us to set up the further research process (esp. the Delphi study), and they provided “theoretical sensitivity” for the entire research process (in the sense of GTM [24]).

The qualitative approach allowed for a flexible research strategy, which was necessary to align with the realities and relevance criteria of the research field [25]. This flexibility was extremely important in our research for two reasons: First, for the reasons described earlier, our research design intended to incorporate a variety of perspectives from the outset. These consistently affected the further direction of the research process, requiring a high degree of openness from everyone involved in the research. Second, the project took place during the COVID-19 pandemic, which brought with it the challenge of flexibly adapting the design to the respective legal, local and institution-related requirements.

**Figure 1:**
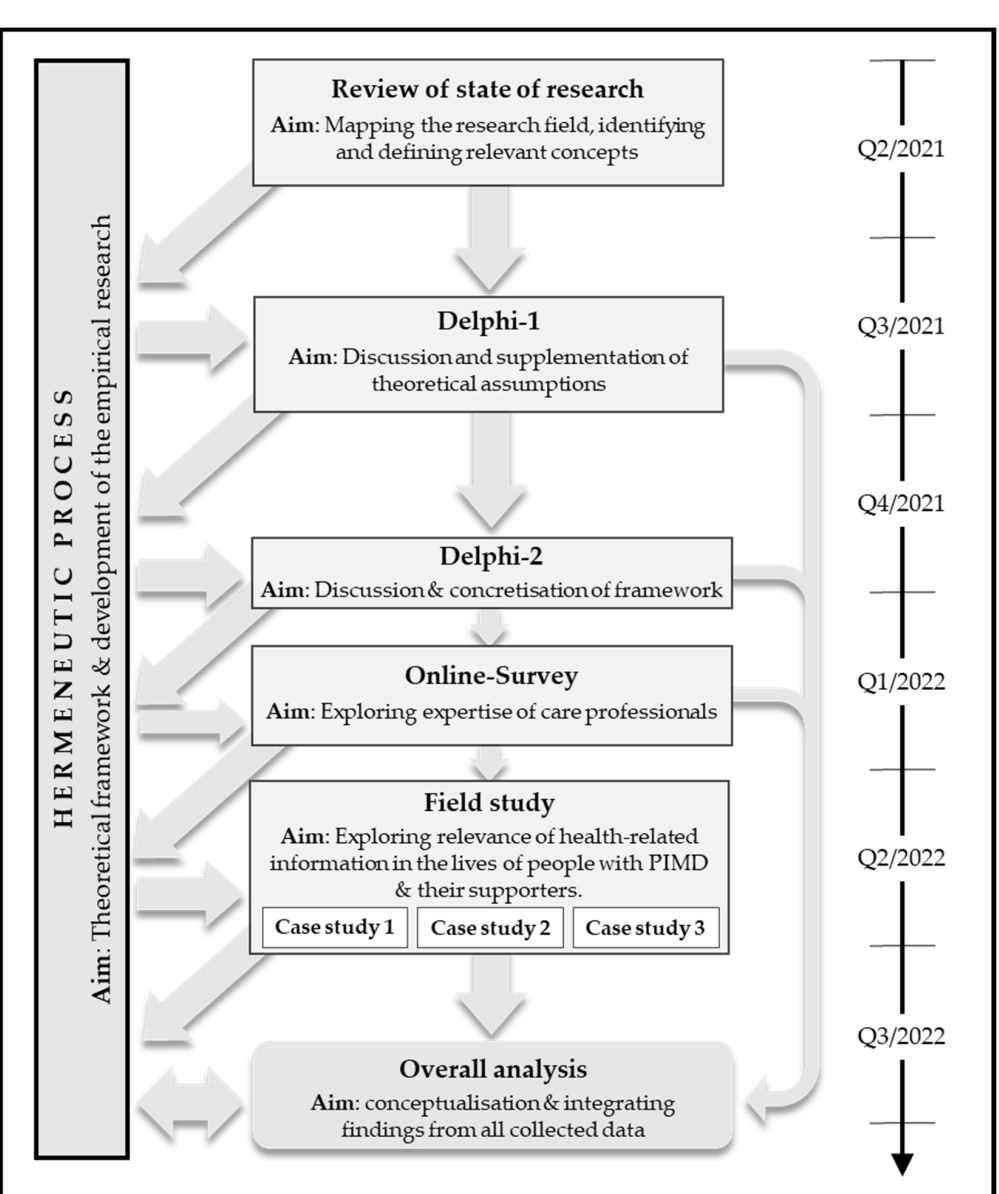
Visualisation of the research process.

Figure 1 provides an overview of our complex, circular and multimethod approach.

In addition, and to increase the comprehensibility of our research process, we include the most relevant survey instruments for our overall analysis as an appendix to this article (see Appendix A).

### 2.1. Delphi Study

Drawing on the results of our initial review of the state of research (Wiki method), we set up a Delphi study [26] with two rounds. The aim of the Delphi study was to discuss and supplement our theoretical findings [27] in order to develop a substantiated yet viable theoretical framework for the research process.

Regarding recruitment, we defined that potential participants should be familiar with the population of people with PIMD and represent one of three perspectives: (1) academia (especially education and health sciences), (2) funding agencies, or (3) service providers. We wrote to 29 people according to these criteria and received 19 confirmations of participation.

**Figure A1:**
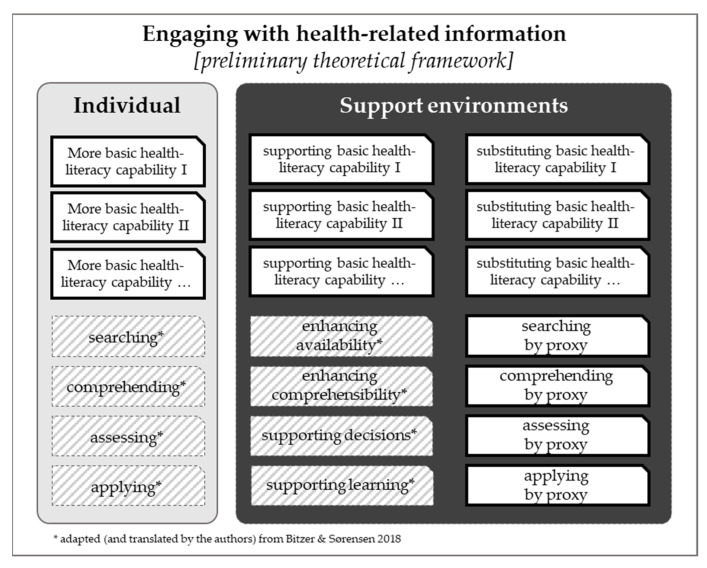
Preliminary theoretical framework resulting from the analysis of Delphi-1.

The first round (Delphi-1) was carried out from 16 July to 30 September 2021 and culminated in 14 experts actually participating. Delphi-1 included 13 open-ended questions on the thematic focuses of the project (see Table A1 in Appendix A for details). We analysed the feedback in an open and axial coding process [22,28] using qualitative coding software (MAXQDA). The results of our analysis contributed to establishing a preliminary theoretical framework (see Figure A1 in Appendix A).

In the second round of the Delphi study (Delphi-2, conducted from 20 December 2021 to 16 January 2022), the main findings of the first round were summarised, and the participants were asked to give their assessment on a Likert scale (they could explain their assessments in additional free-text fields). The feedback of the 11 remaining experts cannot be presented here in detail, but it can be said that our preliminary framework received considerable overall approval. As with Delphi-1, the results of the analysis in round 2 were included in the overall analysis (see Section 2.4).

The preliminary framework that emerged from the two rounds of the Delphi study was driven by conceptual considerations about health literacy responsiveness [29] and included the following elements that guided the further research process (i.e., the online survey and field study): (1) basic health-related capabilities of people with PIMD that can contribute to improving their health literacy, (2) the responsiveness of their support environments, and (3) the substitute, proxy or self-initiated actions with which their support environments undertake health literacy requirements (see Figure A1).

### 2.2. Online Survey with Care Professionals

Based on the results of Delphi I and further research (hermeneutic process, Wiki method), we composed an online survey [30,31] which targeted professionals in disability care services who work with people with PIMD.

The participants were recruited throughout Germany via major professional and disability advocacy associations as well as via large providers of disability care services. Recruitment took place in several phases, and participation was possible for a total of 10 weeks starting 24 January 2022. We expanded our recruitment scope after the first four weeks, as it became apparent that professionals working in residential settings were somewhat underrepresented. Once again, we contacted large service providers and personal networks.

We have succeeded in recruiting a total of 111 care professionals who participated in our online survey. They work mainly in residential settings and sheltered workshops for people with disabilities (see Table 1 for more details). It was quite challenging to identify this target group, as there is no legal definition of the group of people with PIMD. The services they use are generally aimed at a broad group of people, in which people with PIMD represent only one group among many. Therefore, there are no concrete numbers regarding the total number of care professionals working with this group of people. For our research, however, the explicit support of people with PIMD was of particular relevance, so we explained this focus in a detailed cover letter and described it as a selection criterion for participation. Most participants stated that they support people with PIMD very frequently (97 of 111 participants on a daily basis).

In a total of 16 mainly closed questions, the participants were asked about their perceptions of and experiences in communicating (about) health-related information (the questionnaire can be found in Appendix A, see Table A2). The analysis of the material was done in two steps: First, the quantitative data were analysed descriptively and statistically [32], and the free-text answers were analysed using open and axial coding. Second, the results of this step were included in the overall analysis (see Section 2.4). In addition, the findings were decisive for the further research process in that they raised new questions that we were able to explore more specifically in the field study (e.g., about the specific potential of multi-sensory approaches).

### 2.3. Ethnographic Field Study

The field study focused on the exploration of the importance of health-related information in the everyday lives of people with PIMD and their supporters. Therefore, we pursued a multi-perspective approach, following the principles of participation-oriented research [33,34], which aims to maximise participation for those people who are unable to communicate their perspectives and needs without support. Following the guiding principle of this approach, we conducted a research design that allowed us to obtain the individual experiences and needs regarding health-related information and its communication from the participating individuals themselves. The concomitant challenges (little or no verbal language, high level of support, presumably limited cognitive-reflexive skills) were addressed with a multimethod design based on the Mosaic Approach [35]. We developed individualised survey methods and instruments that included elements of Photo Voice [36], participant observation [37], but also innovative approaches, which are outlined below (2.3.1). Furthermore, the aspect of informed or ongoing consent is and was of overarching importance in this participatory approach (2.3.2.) which entails continuous (ethical) reflection on the part of the researchers (2.3.3).

#### 2.3.1. The Case Studies

The ethnographic field study comprises three case studies, each consisting of a person with disabilities and their respective circle of support. Two case studies were conducted to get insights into the occurrence, meaning and communication of health-related information in the everyday lives of people with PIMD. The sample was constituted through an open enquiry to various organisations of disability care facilities. Inclusion criteria were the diagnosis of a profound disorder of intellectual development (6A00.3 in ICD-11), non-verbal communication, full legal age and a comprehensive need for support in everyday life. The third case study was conducted in a participation-oriented way with three persons with ID (and verbal language competencies) and aimed to obtain knowledge about the relevance of health-related information in assisted living settings by focussing on structural perspectives. Due to the individual design of each case study, our approaches in the field studies are not easily replicable. However, in order to make the procedure comprehensible, all three studies are described in detail below.

Case study 1 involved a young man with PIMD in a sheltered workshop. He has no verbal language, is very restless in terms of his mobility and needs extensive support in most activities of self-care. In order to receive more information on his individual preferences and needs and his communicative possibilities of showing agreement and disagreement, we first sent out a brief questionnaire to his (formal and informal) caregivers. Based on this information, we prepared for our first meeting and, more importantly, the possibility of informed consent to participate. For this purpose, we created a multimedia and multisensory research cloth that allowed active engagement with health-related information and addressed different sensory modalities according to the participant’s preferences (tearing off objects and listening to a song). This cloth was also used in the further research process to indicate that we would share the (research) day with him in the sense of ongoing consent. On two days, participant observation was conducted [38] to investigate the relevance and occurrence of health-related information in the participant’s everyday life. These findings were consensually documented in observation protocols, and some situations were videotaped. In addition, two semi-structured interviews were conducted with two of his professional caregivers. This enabled us, on the one hand, to clarify our open questions and uncertainties of the observations and thus also increase their validity. On the other hand, we were also able to collect the perspectives and experiences of the participating care professionals regarding the communication of health-related information.

Case study 2 took place in an inclusive residential setting where a young woman with PIMD lives with ten disabled and non-disabled housemates. The study participant communicates exclusively by body language and has additional comorbidities due to tetraplegia, which affects mobility (she uses a wheelchair that she cannot operate on her own) and most functions of the digestive and metabolic systems. Consequently, she is dependent on comprehensive long-term care.

As with case study 1, we first sent out a questionnaire to her supporters asking about her needs, preferences and communication possibilities. Again, these findings helped to create an individualised approach. For this case, we developed a “multisensory research box”, which invited the participant with PIMD and her supporters to explore health-related situations and topics in a multisensory way and to “record” the spatial-sensory interactions that are important for her [39]. The research box contained an instant camera, which enabled the study participant and her supporters to capture significant situations in the context of health-related information. There were also zip-lock bags and plastic boxes to collect health-relevant items such as olfactory evidence (liquid on a cotton pad). A recording device made it possible to capture auditory impressions. Special documentation sheets were also provided, requesting the co-researching supporters to outline the situation and context in which the photo was taken or in which the collected object became relevant. The box remained in the group home for the period of four weeks and was opened together with all participants at a further research meeting. The collected objects and sensual impressions, as well as the photos, were viewed and contextualised in a joint “tour” of the individual (research) locations. We were allowed to videotape this joint exploration of her everyday life. Furthermore, in a final group discussion with the participating care professionals, we discussed general questions regarding the communication and importance of health-related information with people with PIMD.

Case study 3 focused primarily on a structural perspective and explored the question of which structural conditions affect the communication of health-related information in assisted living. In this case, we developed a participation-oriented approach with residents of a decentralised group home. At an initial meeting, the project was presented to the entire group of inhabitants, whereupon three individuals expressed interest in participating in the research. The three participants communicate verbally, and some also have written language skills. Thus, we informed them about the research project and its objectives verbally and we also provided a leaflet in easy-to-understand language. Over a four-week period, they used the Photo-Voice method [36] to document situations in their everyday life that represented their perspective regarding health-related information. These findings were contextualised in a group discussion and jointly categorised in a mind map. The results of this case study generated insights that focused on structural aspects of disability care services, and as such, they were extremely relevant for the contextualisation of the other two case studies. They also highlight how multi-perspective research approaches can contribute to broadening the view and adequately reflecting the complexity of the research field.

#### 2.3.2. Informed or Ongoing Consent

People with PIMD are considered incapable of giving consent [40], which is why in our research, we also obtained the consent of their legal guardians. However, the denial of individual decision-making and consent capacities has long since ceased to correspond to scientific and ethical consensus [41]. Thus, participatory research calls for providing opportunities for this group of people to participate in research processes from the very start [41,42]. People with PIMD can certainly indicate whether they agree or disagree with participation in a research project. However, researchers must be particularly sensitive in this regard. By also taking into account the perspectives of the (professional) caregivers, steps must be taken to both: inform the person with PIMD about the research project and ensure consent by the person with PIMD him/herself. In the present project, we developed a checklist for the realisation of participation-oriented research with people with PIMD [43] for this purpose. This checklist requires the researchers to be ethically sensitive in all phases of the research process by means of reflection questions. At the same time, it ensures that the person involved can always renegotiate his or her consent in the sense of ongoing consent [44] throughout the entire process.

#### 2.3.3. Reflexivity as a Basic (Ethical) Attitude

A research design such as the one described here challenges all participants to break new ground and maintain a high degree of reflexive sensitivity. This brings with it the challenge of having to adapt the design situationally and individualise the entire research process in a consensual manner. It also includes complying with anthropological-ethical principles at every step, to adequately address the vulnerability of the group of people. Appropriate documentation (interview protocols, work with the developed checklist, elaborated participation-oriented research materials) must be carefully provided and represents an essential feature of the reflective research process.

### 2.4. Overall Analysis

As outlined above, our research involved different methodological approaches, which were also reflected in different methods and phases of analysis.

In order to enable an overall (cross-case and cross-data) analysis of the data, grounded theory [22] was also used throughout the process. Thus, we further developed our preliminary framework that emerged from the Delphi study. In the sense of axial and selective coding, we reviewed, adapted and supplemented the existing categories and examined the relationships between them.

In this way, a coding system was created with which all three project goals (see 1.) could be put into perspective. For the present contribution, the focus is on the second objective, “Exploring the relevance and accessibility of health-related information for persons with PIMD and their supporters in the context of the pandemic and beyond”. To illustrate our evaluation process and to provide insight into our data, the relevant excerpt from our codebook can be found in Appendix B (see Table A3). The entire document is available upon request. This complex process of data analysis enabled us to derive overarching insights into the relevance of health-related information as well as the communication and design of this information. These findings are outlined in the following presentation of our results and then discussed against the background of existing health literacy conceptualisations.

## 3. Results

We present below the central categories of the overall analysis of our data (for an overview of the relevant categories and subcategories, an excerpt from our codebook can be found in Table A3 in Appendix B). We will first approach health-related information from the perspective of people with PIMD and their capabilities (3.1) and then shift the focus to the role of the social context: in particular, to care professionals (3.2). In the third step, we will present our findings on intersubjective engagement with health-related information (3.3).

### 3.1. Basic Health-Related Capabilities of People with PIMD

In this section, we present two categories of our overall analysis, under which we have collected all evidence dealing with the basic health-related capabilities of people with PIMD. First, we will look at how people with PIMD engage with health-related information and requirements (3.1.1). Then we will focus on the basal communication capabilities that are important when engaging with health-related information (3.1.2).

#### 3.1.1. Basal Engagement with Health-Related Requirements by People with PIMD

People with PIMD are a very heterogeneous group of people with different capabilities with regard to engaging with health-related information and requirements. We have identified three such capabilities in our data, which we will now present (please also consult the codebook in Appendix B, i.e., 1.3 in Table A3): (1) the capability of realising changes in one’s own health, (2) the capability of contextualising health-related requirements in the life course, and (3) the capability of comprehending the relevance of (more abstract or long-term) health-related information.

A fundamental capability related to engaging with health-related information is the ability to perceive changes in one’s own health situation. On the one hand, this refers to situations in which the individual is confronted with sudden health requirements (see also subcategory 1.2.1 in Table A3), e.g., sudden pain. The individual then has a direct interest in dealing with the respective “pressing” health issue. Confronting and dealing with pain and discomfort is a particularly plausible example and was also a recurring theme in Case Study 2. On the other hand, changes in one’s own health can also only occur after a longer period of time, e.g., when recovery slowly sets in after a long period of illness or discomfort. In our field study, we have found evidence that people with PIMD can also perceive such long-term health changes as the following statement of a care professional illustrates:

“*Because she sits in the wheelchair so much, it is unavoidable that she simply gets back pain. And we have already tried out different positioning techniques, but I would say that I don’t have the impression that [the study participant] immediately understands the moment we change her position: ‘Ah yes ok, that’s what they are doing now, so that my back pain stops.’ As [my colleague] said, this comes later, when she realises: ‘Ah, okay, now I feel better’. And then you see her smile again and all is well with the world.*”

(case study 2_interview with caregivers, pos. 10)

Even if they may not always be able to communicate it to their support environments, people with PIMD may be able to perceive health changes. Another related capability is the ability to contextualise changes in one’s own health or other health situations and requirements in one’s life course. In the overall analysis, we assigned statements or observations to this subcategory that indicate that people with PIMD recognise health issues. Dealing with menstrual cramps or the need for food are particularly obvious examples that also came up in our field study. Moreover, due to their vulnerability to additional health conditions, people with PIMD are, more than others, familiar with a variety of health situations and requirements. For example, due to additional physical impairments, they might regularly need to use aids to mobilise their limbs (Case Study 2), or they might be able to show others how to handle their incontinence pants (Case Study 1).

The third subcategory and basic health-related capability of people with PIMD that we were able to identify in the data is the capability to comprehend the relevance of health-related information and the potential impact it can have on one’s own health situation. A number of participants in the Delphi study and in the online survey pointed out that this is the most demanding capability, especially when it comes to abstract or long-term information whose relevance is not immediately obvious to people with PIMD:

“*From my point of view, (health-related) information should always be relevant and integrated into everyday life, tied into concrete situations such as care settings or meals. In the crisis/pandemic health-related information was/is of less relevance (to people with PIMD) than the specific protective measures, which can be experienced physically and concretely*.”

(Delphi-1_LAIE3N, pos. 18)

“*For some people (with PIMD), the whole subject around the pandemic is not tangible; moreover, some are also unable to express themselves in any way or reflect on whether the information we communicate has been understood at all*.”

(online survey_173, pos. 4)

These two statements can be summarised as follows: A lot of health-related information is too difficult to grasp for people with PIMD since it might be too abstract for them. In the field study, for example, the difficulty of maintaining a healthy lifestyle was addressed several times: In case study 3, the specific issue was the difficulty in comprehending the benefits of a healthy diet, as these are not immediately perceptible (case study 3_interview2, pos. 331–339). For the study participant in case study 2, it was challenging to understand the use of oral hygiene measures (case study 2_transcript of video1).

However, we were also able to find initial evidence that certain sensory stimuli (like smells and sounds) or spatial settings can help people with PIMD better understand upcoming health-related situations or requirements. For example, the study participant in case study 2 can be helped to prepare herself for oral hygiene by smelling a mouth rinse solution (she opens her mouth when smelling it). The same study participant also found it easier to adjust to health-related requirements when she was in her bathroom (which became evident through her irritation when we wanted to explore health-related requirements with her on the balcony of her group home). Multisensory impressions and spatial settings that are associated with certain health-related actions thus seem to have quite an informative character. They can help people with PIMD to better understand or prepare for more abstract health-related issues.

#### 3.1.2. Basal Health-Related Communication Capabilities

The presentation of results so far has repeatedly indicated that people with PIMD want to and are able to participate in communication about health-related issues. These capabilities will now be examined in more detail, drawing on the following three subcategories of our analysis (i.e., category 1.4 in Table A3): (1) communicating one’s own health needs, (2) communicating one’s own need for information, and (3) having a say in the way health-related requirements are implemented.

The first subcategory comprises situations dealing with how people with PIMD communicate their own health needs. The examples above already illustrate how people with PIMD express their health situation or their needs (e.g., the study participant expressing her pain in her mouth). However, our study also shows that it is not always the case that the expressions of people with PIMD about their health needs are understood by those around them, as this statement illustrates:

“*When she’s feeling really bad (…) we talk to her a lot about it, like this: ‘I notice that you are in a bad way right now. [What your caregivers in your sheltered workshop wrote into the notebook] doesn’t read as if you had a bad day, let’s somehow see what we can do about it.’ And since we do a lot of trial and error, she is also annoyed at some point by how long the process takes until she somehow feels better. This often comes to a head. That you simply notice that she is, she wants things to go well for her again. And I’m not in a position to reach that goal quickly and she gets so annoyed about it*.”

(case study 2_interview with caregivers, pos. 52)

This statement illustrates a typical dilemma: people with PIMD do express discomfort or other urgent health needs. However, this does not always provide an indication of the cause or location of the discomfort. People with PIMD may not be aware of this themselves.

This already introduces the second subcategory regarding participation in communication about health-related issues: This is about situations in which people with PIMD express their need for health-related information—or one could also say more simply: the (probably non-verbal) health-related questions that people with PIMD address to their environment. Even if the environment cannot always answer these questions, it seems to us to be an essential achievement in terms of responsive support for the group of people if people with PIMD can express these questions and they can “ask” them to an attentive counterpart.

The third subcategory relates to the communication on how health-related requirements (such as healthcare activities) are implemented. This aspect was particularly visible in case study 2. For example, one caregiver reported that the study participant was reluctant to carry out the necessary physiotherapeutic measures on the exercise bike and could not be motivated to do so. By offering different alternatives that would make this activity more enjoyable for her, a dialogue about her preferences was established until a mutually acceptable solution was found: They now play her favourite music while she uses the exercise bike. In case study 1, the study participant is allowed to do his favourite activity (tearing paper and plastic) after he has completed a COVID-19 test. It should now be clearly observed that finding compromises and maintaining a dialogue about health-related requirements is apparently very important for successful participation in health-related communication.

### 3.2. The Role of the Social Context

In the following, we will shift the perspective from the person with PIMD to the social context, or more precisely: to the support environment. We will look at the question of how care professionals support people with PIMD in their engagement with health-related information. First, we will look at the results that show how care professionals engage with health-related information by proxy (3.2.1). Then, we will present a very central requirement of health-related communication, namely the requirement to illustrate, facilitate and raise awareness of health-related information (3.2.2).

#### 3.2.1. Engaging with Health-Related Information by Proxy

A very central and recurrent finding of the study was that care professionals perform many health literacy-related tasks by proxy in everyday life. We want to illustrate our findings on this by means of four subcategories of our analysis (i.e., category 2.1 in Table A3): (1) assessing health situation and needs by proxy, (2) searching for and assessing information by proxy, (3) passing on and discussing health-related information by proxy, and (4) raising awareness on health-related issues.

It has already become clear in previous excerpts from the data that care professionals are often confronted with the challenge of assessing the health situation of people with PIMD by proxy since they are often unable to make themselves understood. Nevertheless, this kind of attentiveness to the health needs of their care receivers is sometimes of vital importance.

“*Since the people I work with are rarely able to express themselves verbally about their state of health, the question (of how I support people with PIMD to develop an understanding of health-related information) is difficult to answer. Of course, we accompany the people and interpret their actions in relation to their state of health, but they rarely show pain and I cannot assess to what extent they show signs of a corona infection for example or to what extent they understand this information*.”

(online survey_63, pos. 1)

As the statement from the online survey indicates, pain detection and continuous behaviour observation are typical health-related requirements in the everyday support of people with PIMD. This is also mirrored in the case studies of the field study.

We have also already referred to the fact that people with PIMD usually have difficulties in searching for and critically assessing more abstract health-related information themselves. However, much health-related information that is relevant in or for the lives of these people is abstract and difficult to understand. Care professionals take on this role on a proxy basis: During the pandemic, it was mainly they who dealt with COVID-19-specific information on (mostly individually very different) infection risks or prevention measures. The online survey made it clear that in order to meet these requirements, care professionals need a lot of support and guidance. Here we asked them which sources of support they consider helpful in communicating health-related information on the pandemic to people with PIMD (see question B4 in Table A2 in Appendix A). The possibility of talking to health workers (such as doctors or nurses) about individual and specific issues was rated the least helpful of all the options we gave (only 39.6% of the participants agreed or strongly agreed to the helpfulness of this item). However, all other sources of support were rated as helpful by more than half of the participants. On the one hand, there is evidence that communicating health-related information during the pandemic requires a lot of time and professional resources and expertise, especially to convey this information in direct interaction with people with PIMD (54.1% of the participants agree or strongly agree), but also to develop original and individualised information resources and materials (53.1% of the participants agree or strongly agree). On the other hand, easy-to-understand information was also considered helpful for care professionals themselves to communicate with people with PIMD about this content in a customised way (58.5% agree or strongly agree). However, what was rated as particularly helpful were target-group-specific information resources (61.2% agree or strongly agree).

We also asked which of these sources of support they actually used or were able to use, and it turned out that 61.3% of the respondents were able to use easily understandable information to communicate health-related information during the pandemic. Some 55% of respondents stated that they had taken the time (and professional resources) to interact directly with the target group to discuss these issues. Although rated very helpful, only 38.7% were able to use target-group-specific information resources and materials, and only 29 (21%) of the respondents sought exchange with health workers.

In the online survey, we asked a multiple-choice question: how did care professionals find out about health-related information resources during the pandemic (see question C1 in Table A2, Appendix A)? The results indicate that the care professionals who participated in our online survey were or had to be proactive in terms of finding suitable information resources, with 56.7% of the respondents stating that they had done their own research (e.g., on the internet) to obtain such resources. Further to this, 60.4% of the 111 respondents stated that they learned about these information resources through a hint from their professional environment, which implies that there were colleagues who passed on these information resources.

In general, passing on, sharing and discussing health information with others is another very significant requirement that care professionals take on by proxy. This was mentioned, among others, by the co-researchers with ID in case study 3. For example, one interviewee said that the care professionals in his assisted living facility regularly passed on health-related information to health specialists because the people they support cannot do this adequately or at all themselves (case study 3_ interview1, pos. 83–84). In the other two case studies, passing on health-related information was also a recurring theme. For example, in both case studies, notebooks were mentioned in which important health-related information was passed on to other institutions (e.g., previous drinking quantity or reports on sleeping problems of the person with PIMD).

As all the data collected in our research clearly show, correspondence with relatives generally plays a special role in the communication of health-related information. However, perceptions of the constructiveness of the exchange with relatives vary widely: in most cases, the special expertise of relatives is recognised as a valuable source of information (e.g., Delphi-1_WIMA9H, pos. 8 or online survey_243, pos. 6). On the other hand, challenges in the exchange with relatives are also mentioned, such as the taboo of certain health topics in the family environment (online survey_23, pos. 9) or the difficulties of parents to also acknowledge the health-related assessments of care professionals (in case study 2_interview with caregivers, pos. 29–37).

The last health-related requirement presented here, which care professionals often take on by proxy, is the requirement to raise awareness of health-related issues. This is necessary because people with PIMD are unfamiliar with many health issues and therefore need support in learning about new health-related information. This means that care professionals not only provide occasion-related information but also communicate issues that can be relevant to a person’s health beyond concrete situations. In our data, these tasks were mainly described as desiderata. Two participants in the Delphi study, for example, noted that care professionals should also address health prevention measures proactively (Delphi-1_WIVA3R, pos. 8 and Delphi-1_WIMT9N, pos. 18). In case study 2, the need to maintain mobility due to the severe physical impairment of the study participant with PIMD was a topic that the professionals had to repeatedly address on their own initiative (case study 2_documentation sheet3_pos. 5).

This last subcategory already indicates the professional requirement for supporters to illustrate information appropriately in order to facilitate understanding. Our findings on this are discussed in more detail in the next section.

#### 3.2.2. Illustrating and Facilitating Understanding

We have already shown that there is a lack of suitable, target-group-specific information materials. Care professionals are therefore often called upon to either develop original and individualised information resources and materials themselves or to adapt existing (non-target-group-specific) information materials in an individualised way. In the following, we present our results on their approaches to communicating health-related information by (1) considering individual communication modalities, (2) taking into account individual sensory modalities, and (3) giving opportunities to actively engage with elements of the information (i.e., subcategory 2.1 in Table A3).

In the online survey, we asked the care professionals which communicative modalities approach they consider most suitable for communicating health-related information (see question A4 in Table A2 in Appendix A). Only 20.7% of the respondents agree or strongly agree, while 44.1% disagree or strongly disagree that text-based resources in easy language are suitable in this respect. In contrast, 43.2% of the respondents agree or strongly agree that video-based resources are suitable for conveying health information to people with PIMD. Face-to-face interactions using augmentative and alternative communication, resources with illustrations and pictograms and face-to-face verbal interaction were rated similarly suitable (54,9%, 55,8% and 56,1% of the respondents agree or strongly agree with each item, respectively).

We also gave the opportunity to name further modalities, and some of the respondents made use of this. Among other things, the heterogeneity of the group of people was underlined, and the potential of multisensory approaches was addressed. This also prompted us to take a closer look at this in the field studies.

In fact, we identified numerous situations in which multisensory approaches were used. We have already referred to the use of olfactory stimuli in case study 2: The care professionals use these to prepare the study participant with PIMD for certain health-related requirements (in this case, the use of a certain mouthwash solution). Haptic approaches were also addressed or used. For example, in case study 1, the study participant had the opportunity to feel and engage haptically with various everyday health-related objects (e.g., a mask or hygiene gloves) (case study 1_transcript of video, pp 10–11).

In general, we were able to gain some preliminary evidence that indicates that haptic or active engagement with elements of the information can be conducive to the communication of health-related information. In case study 2, for example, the study participant was given the water flosser to adjust to the situation of oral hygiene. In the online survey, the inclusion of everyday life objects was also rated highly practicable (see question C2 in Table A2): 86 of the 111 respondents (77.5%) stated that they use everyday life objects to adapt health-related information in such a way that it is understood by people with PIMD (the only other strategy that was used at a similar frequency was explaining the information in their own and easily understandable answers).

### 3.3. Joint Engagement with Health-Related Information

Since people with PIMD share a large part of their day with care professionals, both care receivers and caregivers are often confronted with health situations that neither of them can immediately comprehend. Exploring these situations together, communicating about them, jointly finding health-related decisions as well as working together to carry out health-related requirements are common demands that affect both people with PIMD and their supporters and require their mutual commitment. In the following, we will present our findings on such situations or requirements of joint engagement with health-related information (see category 2.3 in Table A3).

Especially at the beginning of the COVID-19 pandemic, everyone was confronted with a high degree of uncertainty. This is illustrated by the following statement from a study participant with an intellectual disability:

“*So, there were rules and that was very much. There were new changes all the time, and you couldn’t keep track of them at all. Even the caregivers were overwhelmed by paperwork. (…) That was quite difficult, yes, how do you have to behave*.”

(case study 3_interview2, pos. 22)

The statement demonstrates that people with disabilities and caregivers alike were challenged by a dynamic information situation. Later in the course of the interview, the study participant elaborates on how both had to learn what the acute threat was, how to protect themselves from infection, etc. Additionally, in everyday life (beyond the context of the pandemic), situations often arise that are not easy to keep track of. Especially when pain or discomfort occurs suddenly and unexpectedly, everyone involved is challenged to deal with the lack of information. It is then of great importance to search together for the causes and possible treatment methods.

Exchange and dialogue on an equal footing form an essential basis for successful communication in cases of mutual non-understanding. If verbal language is not an option, communication through physical communication (in the sense of intercorporeal communication) can be helpful. A care professional from case study 2 describes this in a particularly impressive way:

“*So when (the study participant) is really in pain, you can see and feel it. It really goes through my spine, too. It’s really like that, I notice a difference whether it’s perhaps such discontent or really extreme pain.*”

(case study 2_interview with caregivers, pos. 51)

What the care professional describes here refers to the often instinctive or spontaneous nature of communication. Especially in situations with an acute urge to act, communication can also do without verbal language.

Therefore, it becomes clear that a high degree of responsiveness is required from both care receivers and caregivers when communicating about health-relevant situations or issues. This means that caregivers are required to be attentive to the signals and needs of people with PIMD. In addition, it also requires that they recognise people with PIMD as equal partners in communication who want to and can participate in communication about health-related issues and decisions that affect their own lives.

## 4. Discussion

The aim of this article was to present our findings on how people with PIMD and their supporters engage with health-related information. The results presented here could provide a wide range of insights in this regard. We will first summarise and synthesise our findings (4.1) and then discuss them against the background of existing research and studies in health literacy research (4.2). In the third section, we will address the limitations of our research (4.3).

### 4.1. Basal Engagement with Health-Related Information

**Figure 2:**
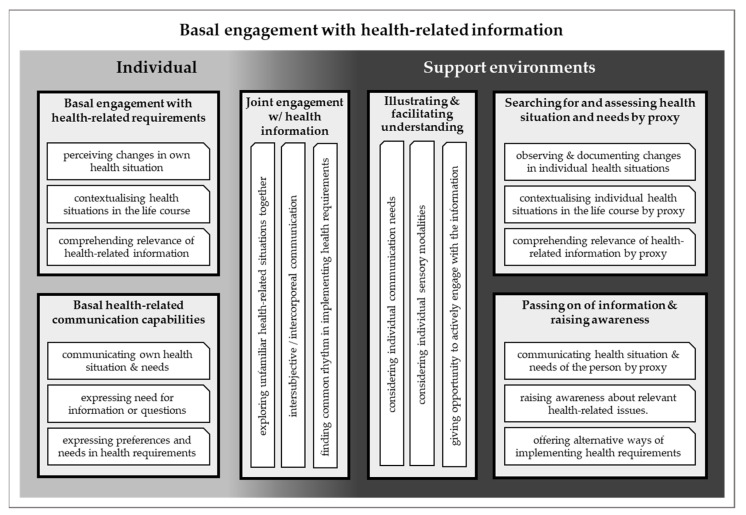
Visualisation of our findings on the basal engagement with health-related information.

In this section, we will consolidate our findings in order to outline our contribution to the conceptualisation of basal engagement with health-related information. Figure 2 provides an overview of our results and is intended as a visualisation of the categories and subcategories of our overall analysis.

We will now first summarise our findings on the basic health literacy capabilities of people with PIMD (4.1.1). Then, in 4.1.2, we will shift the perspective to their supporters and summarise our findings on their contribution regarding engagement with health-related information.

#### 4.1.1. Basic Health Literacy Capabilities of People with PIMD

We have shown that people with PIMD are confronted with a lot of health-related requirements and issues that are not graspable for them on a daily basis. On the other hand, we have also been able to show that people with PIMD do have a variety of health-related capabilities. They are capable of engaging with health-related requirements by:

- Perceiving changes in their own health situation;
- Contextualising health situations in the life course;
- Comprehending the relevance of health-related information and the potential impact they can have on one’s own health situation.

They are also capable of participating in health-related communication by:

- Communicating one’s own health situation or needs;
- Expressing health-related questions;
- Expressing preferences and needs in health-related requirements.

In the following, we want to summarise these capabilities under the term “basic health literacy capabilities”. They are “basic” because they are elementary and fundamental for engaging with health-related information and the development of health literacy (not only for people with PIMD). Since these capabilities are essential to gain better orientation in dealing with health information, they are, therefore, significant facilitators of health literacy, albeit very basic ones. Following the philosopher Martha C. Nussbaum [45], we use the term “capabilities” to emphasise that engagement with health-related information is not only a matter of individual skills and abilities but also depends on opportunities to (actively) engage with health-related information.

The basic health literacy capabilities that we were able to identify in our research are, of course, not comprehensive, as they only represent the results of our largely explorative study. However, they may illustrate that, at a closer look, people with PIMD can contribute much more to the communication of and on health-related information than commonly assumed—in a multitude of situations, every day.

#### 4.1.2. The Role of the Support Environments

Furthermore, while these capabilities are very basic in nature, there is still a high and complex need for support. Recognising the capabilities mentioned above is an important step, but promoting and fostering them is not always an easy task. However, our results show that fostering basic health literacy capabilities is possible.

By individualising and communicating health-related information, caregivers help people with PIMD to better understand health situations. By using everyday objects and situating the relevant information in the biographical, spatial and social context, they make it easier for people with PIMD to become familiar with health-related requirements. This facilitates recognition of health-related requirements and situations. It is also important to consider the situational relevance of the information when communicating it: Why is the information important in the given situation? How might the information be important in the future, and how can this be made tangible to the person with PIMD? If these questions are answered sufficiently, people with PIMD can also be supported in accessing and understanding the relevance of health-related information.

Finally, the support environments enable participation in health-related communication through a responsive attitude and the use of different communicative approaches that address other senses besides the visual.

### 4.2. Discussion of the Results

In the following, we will discuss our findings against the background of previous health literacy research. Again, we will first approach this from the perspective of people with PIMD and our findings on their needs and capabilities (4.2.1) and then draw on our findings on the role of the social context (4.2.2).

#### 4.2.1. Health Literacy and “Basic” Capabilities

Various publications and studies pay attention to the discrimination of people with intellectual disabilities (ID) in health literacy research (see also Section 1.2 in this article). The criticism is primarily concerned with the focus on individual, functional or cognitive aspects. With regard to health literacy among people with profound intellectual and multiple disabilities, this criticism must be particularly emphasised: As our findings show, people with PIMD are usually unable to meet conventional health literacy requirements without support, as these presuppose communicative [15] and cognitive skills [12,16,46,47] that they usually do not possess. Even the provision of information in easy language is absolutely insufficient here [15,16].

However, our findings illustrate that it can be rewarding to broaden the view and to start in particular with those abilities that are often assumed but thus overlooked. Coping with health-related situations is an essentially active process of emotional, bodily and communicative engagement with health-related information and requirements. Even though reflective and abstract thinking is required for many health-related tasks, these supposedly “higher” cognitive processes are grounded in active and bodily engagement with the world. Therefore, these are fundamental for the (further) development of individual health literacy.

#### 4.2.2. Health Literacy and the Social Context

In health literacy research, there is a growing awareness of the impact of the social context, which has led to an increasing focus on interactive health literacy concepts [48]. The acknowledgement that environments substantially contribute to the development of individual health literacy has led to a lively discussion in recent years about the responsiveness of health systems, which can be bundled under the concept of “health literacy responsiveness” [49]. Such considerations have also recently found their way into the design of disability care facilities [50]. In addition, the need for a responsive environment has been emphasised many times, especially in relation to people with (profound) intellectual disabilities who do not communicate their needs verbally [15,51,52].

Our findings clearly highlight the need for a responsive and engaging environment. As our findings show, people with PIMD rarely engage with health-related information by themselves. On the contrary, they rely on comprehensive support for almost all health-related needs, such as dealing with health-related information, finding explanations for hard-to-grasp health-related situations or communicating about other health-related issues. In addition, our findings also made it clear that the support environments bear a great responsibility for meeting all these requirements. Finally, we were able to make visible the potential of intersubjective processes for understanding and communicating health-related issues [53]. Following Bittlingmayer and Sahrai [46], it can be emphasised that increased consideration of intersubjective perspectives can help to better map the complex interactions between the subject and his or her social, material, and spatial environment in health literacy research.

### 4.3. Limitations

The presented research followed a qualitative, explorative approach. As described in more detail in Section 2, we investigated the research questions using different data collection methods and, especially in the field study, with very individualised research approaches. This represented a great added value for the involved study participants with PIMD because we were able to develop individualised approaches that flexibly and situationally addressed their respective learning and communication needs.

However, this is also a limitation of the research. Especially in the field study, the replicability is limited because the three designs were developed gradually by continuously reflecting the respective situation of the persons involved.

Due to the limited number of participants, the generalisability of our results is also limited. Furthermore, we were not always able to achieve “theoretical saturation” in the selective coding of some categories (especially in subcategories 1.2.3 and 1.3.3, see Table A3) [22]. Therefore, gaps became visible in the overall analysis of our data, which actually required further data collection. The results, therefore, only offer first insights into a field of research and practice that needs to be further explored. Our various studies offer methodological as well as conceptual starting points on which further research can now be based.

## 5. Conclusions

The relevance of health-related information in support of people with PIMD has certainly become visible. Our research has approached the health literacy of people with PIMD from different perspectives and thus generated important insights and future requirements regarding further research activities (5.1), the conceptualisation of health literacy (5.2) and target-group-specific health literacy support of people with PIMD (5.3).

### 5.1. Conclusions for Health Literacy Research

- Our research could show that people with PIMD can be included in research processes through participation-oriented research approaches.
- Since people with PIMD can participate more effectively when different senses are involved, multimethod and multimodal approaches should also be given more consideration in research with this group of persons (cf. case studies 1 and 2).
- A great added value could be achieved by including the supporters in the research process, who are thus not (“only”) included as representatives but as co-researching experts of the social environments of people with PIMD.
- Another lesson learned is the importance of multi-perspectivity. Here it turned out to be very fruitful to recognise self-advocates as life-world experts and to include them in research processes. This can include, for example, flatmates or colleagues of people with PIMD (cf. case study 3).
- In conclusion, health literacy research can benefit greatly from the inclusion of diverse, possibly also conflicting perspectives, especially if it aims at adequately reflecting the complexity of the interactions of different (e.g., individual, structural, social) conditions.

### 5.2. Conclusions for Conceptualisation of Health Literacy

- Health literacy concepts should recognise that focusing on visual and symbolic information proves insufficient for a lot of people whose health literacy is considered low. Not only people with PIMD but also people with dementia, for example, would certainly benefit from multisensory approaches.
- Furthermore, our results underline the importance of the social context. In many cases, it is the (professional) caregivers of people with PIMD who deal with health-related information and communicate (about) this information to (or with) people with PIMD.
- Hence, health literacy must be understood as a responsive and intersubjective concept. Our findings show that in many health-related situations, people with PIMD and their supporters are challenged to explore together what information is relevant and what action is required.

### 5.3. Conclusions for Target-Group-Specific Health Literacy Support of People with PIMD

- The project shows that there is a great need for further knowledge about the way people with PIMD learn best. On the one hand, this concerns the design and communication of health-related information. However, it can also be transferred to all other contents and contexts. Knowledge about educational processes for this group of people is still a great desideratum. This needs to be addressed through further education and training of staff, but also through further conceptual developments.
- Conceptual developments are absolutely necessary in order to support professionals in intersubjective mediation. Here it is necessary to develop “tools”, which on the one hand, contain basic and transferable possibilities and, on the other hand, enable individual adaptation so that it is not necessary to start again and again from scratch in every context and with every person.
- There is a need for further developments to make health-related information accessible for people with PIMD beyond the situational context. Our results show that people with PIMD can participate in communication about a variety of health-related issues, but suitable approaches to communicate more abstract or long-term health issues are lacking.
- In this regard, there is also an urgent need for materials that go beyond visualisations and written language. These materials must convey knowledge multimodally and creatively in different ways. Multisensory stories, for example, are suitable for this purpose. We are currently developing those approaches in our project.

## Figures and Tables

**Table 1:** Sample of the online survey.

	Frequency in	
	*n*	%
Main professional activity of respondents		
Residential home	18	16.2
Sheltered workshop	76	68.5
Daycare facility	2	1.8
Health sector	7	6.3
School	1	0.9
Other	7	6.3
No answer	0	0
Total	111	100
Frequency of supporting People with PIMD		
Never	0	0
Once to three times a month	3	2.7
Up to twice a week	10	9.0
Daily	97	87.4
Others	1	0.9
No answer	0	0
**Total**	**111**	**100**

## Data Availability

The data presented in this study are available on request from the corresponding author. The data are not publicly available due to privacy restrictions.

## Appendix A

**Table A1:** Questionnaire for the first round of the Delphi study (“Delphi-1”; time frame for completion: 16 July to 30 September 2021).

Thematic Focus		Question
Vulnerabilities, communication needs and health literacy of people with PIMD (in general)	A1	*In your experience and view, what vulnerabilities are evident among people with PIMD?*
	A2	*In your experience and view, which communicative needs and particularities are decisive for the group of people with PIMD?*
	A3	*What role does health-related information play for people with PIMD and to what extent do you think there are particularities compared to other people? How do these particularities manifest themselves?*
Vulnerabilities, communication needs and health literacy of people with PIMD in the pandemic	B1	*What particular vulnerabilities were or are evident in people with PIMD in the Corona pandemic?*
	B2	*In which situations and contexts did or do these particular vulnerabilities show up?*
	B3	*What special communicative needs and characteristics were or are evident in people with PIMD in the pandemic?*
	B4	*In which situations and contexts did or do these special communicative needs become apparent?*
	B5	*In which situations and contexts did and does health-related information become relevant for people with PIMD and their supporters in the pandemic?*
	B6	*What specific health-related information was and is relevant in these situations and contexts?*
	B7	*In relation to the health-related information you have just identified: What specific communicative needs arise(d) and result(ed) from this?*
Experiences and ideas for a “better support practice”	C1	*How were the vulnerabilities of people with PIMD that you described earlier addressed during the pandemic? How could these be addressed in the future?*
	C2	*How were the communication needs of people with PIMD that you described earlier addressed during the pandemic? How could these be addressed in the future?*
	C3	*How was health-related information for people with complex disabilities communicated during the pandemic? How could this be done in the future?*

**Table A2:** Questionnaire of the online survey (time frame for completion: 24 January until 3 April 2022).

	Question		Response Format
Health-Related Information in the Lives of People with PIMD (in General)			
A1	*Based on your experience, how would you rate the following statements regarding your support of people with PIMD in engaging with health-related information?*		
	A1.1	*I support people with PIMD to understand and contextualise their own health situation.*	*Never—Rarely—Occasionally—Often—Always—N/A*
	A1.2	*I support people with PIMD to communicate their own health situation to others.*	
	A1.3	*I support people with PIMD to seek or obtain health-related information.*	
	A1.4	*I support people with PIMD to understand health-related information.*	
	A1.5	*I support people with PIMD to assess health-related information.*	
	A1.6	*I support people with PIMD to make and implement health-related decisions.*	
A2	*If possible, please tell us (by way of example) how you carry out the above support activities?*		*free text*
A3	*In your experience, which of the following factors play a role in successfully communicating health-related information to people with PIMD?*		
	A3.1	*Time resources*	*multiple choice*
	A3.2	*Professional resources (e.g., special qualifications in the team, …)*	
	A3.3	*Institutional resources (e.g., structured discussion with colleagues, further training, …)*	
	A3.4	*Quality of the relationship with person with PIMD (e.g., duration and intensity of contact).*	
	A3.5	*Personal attitudes of staff (e.g., on health-related topics)*	
	A3.6	*Personal resources and needs of people with PIMD (e.g., individual skills or attitudes, …)*	
	A3.7	*Other*	*free text*
A4	*In your experience, what is the best way to communicate health-related information to people with PIMD?*		
	A4.1	*By providing text-based resources in easy language*	*strongly disagree—disagree—neutral—agree—strongly agree—N/A*
	A4.2	*By providing resources with illustrations, pictograms and symbols*	
	A4.3	*By providing video-based resources*	
	A4.4	*Face-to-face verbal interaction with people with PIMD*	
	A4.5	*Face-to-face interaction using augmentative and alternative communication*	
	A4.6	*Combined approaches*	
	A4.7	*Other*	*free text*
Health-Related Information in the Pandemic			
B1	*How often have you and/or your colleagues used the following approaches to communicate health-related information to people with PIMD in the pandemic?*		
	B1.1	*I provided text-based resources in easy language*	*Never—Rarely—Occasionally—Often—Always—N/A*
	B1.2	*I provided resources with illustrations, pictograms and symbols*	
	B1.3	*I provided video-based resources*	
	B1.4	*Face-to-face verbal interaction with people with PIMD*	
	B1.5	*Face-to-face interaction using augmentative and alternative communication*	
	B1.6	*Combined approaches*	
	B1.7	*Other*	*free text*
B2	*What was the content of the health-related information on the pandemic situation that you communicated as indicated above?*		
	B2.1	*Course of the disease and symptoms*	*multiple choice*
	B2.2	*Routes of infection and spread*	
	B2.3	*Hygiene rules (washing hands, wearing a mask, etc.)*	
	B2.4	*Everyday rules (e.g., distance rules, 2G/3G, contact restrictions)*	
	B2.5	*Corona testing*	
	B2.6	*Corona vaccination*	
	B2.7	*Isolation and quarantine measures*	
	B2.8	*Other*	*free text*
B3	*Which of the following sources of supports could you draw on to provide pandemic-specific health-related information to people with PIMD?*		
	B3.1	*Easy-to-understand health-related information resources on the basis of which this information can be adapted for people with PIMD*	*multiple choice*
	B3.2	*Professional exchange with health workers on individual and specific health-related issues*	
	B3.3	*Provision of target-group-specific information resources & materials for people with PIMD by institutions (service provider, associations, …)*	
	B3.4	*Enough time and professional resources to create original and individualized information resources, materials or conceptual approaches*	
	B3.5	*Enough time and professional resources for more personal interaction with people with PIMD on health-related issues*	
	B3.6	*Other*	*free text*
B4	*Which of the following sources of supports did you find helpful or would you have found helpful?*		
	B4.1	*Easy-to-understand health-related information resources on the basis of which this information can be adapted for people with PIMD*	*strongly disagree—disagree—neutral—agree—strongly agree—N/A*
	B4.2	*Professional exchange with health workers on individual and specific health-related issues*	
	B4.3	*Provision of target-group-specific information resources & materials for people with PIMD by institutions (service provider, associations, …)*	
	B4.4	*Enough time and professional resources to create original and individualized information resources, materials or conceptual approaches*	
	B4.5	*Enough time and professional resources for more personal interaction with people with PIMD on health-related issues*	
B5	*Can you think of any other sources of support not listed above that were not available to you, but would have been or could be helpful?*		*free text*
Health-Related Information Resources and Materials in the Pandemic			
C1	*If you have made use of existing pandemic-specific health-related information resources or materials: How did you become aware of these information services and materials?*		
	C1.1	*Through a hint from a colleague or by my service provider.*	*multiple choice*
	C1.2	*Through public institutions (e.g., funding agencies or health authorities)*	
	C1.3	*Through an association (e.g., welfare organization, charities, …)*	
	C1.4	*Through a hint from my private environment*	
	C1.5	*I did not use existing information resources or materials*	
	C1.6	*Through my own research*	
	C1.7	*Other*	*free text*
C2	*How did you adapt the existing information resources and materials in order to use them for communicating the respective content to an individual with PIMD?*		
	C2.1	*None, this usually worked without any adaptations*	*multiple choice*
	C2.2	*By explaining the content in my own words*	
	C2.3	*By using AAC technologies and aids (bodily forms of communication, non-electronic and electronic communication aids)*	
	C2.4	*By embedding the content in concrete everyday situations (e.g., through role play)*	
	C2.5	*By including real or everyday objects (e.g., mask)*	
	C2.6	*Other*	*free text*
C3	*If the information resources and materials you used are freely accessible, we would be pleased if you would share them with us—either by link or by email*		*free text*
Closing Question			
D1	*Do you have any further comments on the significance of health-related information in the lives of people with PIMD that you would like to share with us?*		*free text*

## Appendix B

**Table A3:** Excerpt from codebook for overall analysis (focus on the categories & subcategories of relevance for this paper).

Category	Subcategory	Definition	Main Data Sources	Exemplary Codes
1. HEALTH-RELATED INFORMATION IN THE EXPERIENCES OF PEOPLE WITH PIMD				
1.1 Health-related situations of relevance for p w/PIMD				
	1.1.1 Health-related situations, in general	General health-related contexts, situations and/or issues that were/are or might become relevant for people with PIMD	Delphi I Field study	*pain; health-promoting lifestyle; injuries; medication; oral and dental health; …*
	1.1.2 Health-related situations in the pandemic	Pandemic-specific and health-related contexts, situations and/or issues that were/are or might become relevant for people with PIMD	Delphi I Field study Online survey	*social distancing; vaccination; infection routes and course of the disease; results from online-survey (C2); …*
1.2 Health-related requirements of relevance for p w/PIMD				
	1.2.1 Health-related requirements with pressing urgency	This subcategory gathers evidence on health-related contexts, situations and/or issues that immediately affect people with PIMD, and require immediate action. They are subjectively and situationally highly relevant and the individual is highly motivated to overcome these situations. Overlaps with subcategory 1.3.1 are expected.	Field study	*(acute or chronic) pain; urgent physical needs; …*
	1.2.2 Regular health-related requirements	This subcategory gathers evidence on health-related contexts, situations and/or issues that affect individuals with PIMD on a regular basis. They are characterised by a dynamic urgency structure, i.e., they only require action when the need is very urgent. They are familiar to the subject, and depending on the urgency, they are subjective and situationally (partly) relevant. Overlaps with subcategory 1.3.2 are expected.	Field study	*(increasing or decreasing) need to eat; (increasing or decreasing) need to empty bladder; …*
	1.2.3 Long-term or more abstract health-related requirements	This subcategory gathers evidence on health-related contexts, situations and/or issues that do not immediately affect the individual but could potentially affect him or her (e.g., in the future). Without appropriate information, these requirements might not appear to be subjectively relevant to people with PIMD. Overlaps with subcategory 1.3.3 are expected.	Field study	*maintaining healthy life-style; understanding measures of disease prevention; …*
1.3 Basal engagement with health-related requirements by p w/PIMD				
	1.3.1 Perceiving changes in own health situation	Basic capabilities of perceiving changes in one’s own health situation or to become aware of “unusual” health situations. This subcategory refers to health-related situations that become (immediately or on a long-term base) relevant to the person (cf. category 1.2). Overlaps with subcategories 1.1, 1.2.1, 1.2.3 and 1.3 are expected.	Field study	*perceiving sudden onset of pain; becoming aware of (gradual) improvement in one’ s own health situation; …*
	1.3.2 Contextualising health situations in the life course	Basic capabilities of recognising certain health-related situations and requirements. The main focus in this subcategory is on capabilities of people with PIMD to contextualise “unusual” health situations (such as illnesses) in their life course. Therefore, past (and perhaps unconsciously memorised) health-related experiences are also taken into account. Overlaps with subcategories 1.1, 1.2.2 and 1.3 are expected.	Field study	*learning to deal with menstrual cramps; recognising symptoms of an illness; …*
	1.3.3 Comprehending the relevance of health-related information	Basic capabilities of understanding and comprehending health-related information that may not immediately affect the individual. Overlaps with subcategories 1.1, 1.2.3 and 1.3 are expected.	Field study	*(difficulties to) understand the use of a face mask; recognising smell of mouth-rinse solution; …*
1.4 Basal health-related communication capabilities of p w/PIMD				
	1.4.1 Communicating one’s own health needs	This subcategory gathers evidence about how people with PIMD express their own health situation or their needs; this includes the opportunities (that they have or that they are given) to address their own health issues to others. Overlaps with subcategory 2.2.1 are expected.	Field study Delphi 1 Online survey	*expressing pain through facial expressions; expressing desire for personal hygiene by eye pointing to bathroom; results from online-survey (A1 & A2); …*
	1.4.2 Communicating one’s own need for information	This subcategory gathers evidence about how people with PIMD express their irritation (resp. “questions”) about health-related situations or requirements; this includes the opportunities (that they have or that they are given) to address their irritation/“questions” to others. Overlaps with subcategory 2.2.1 are expected.	Field study Delphi 1 Online survey	*expressing distress or irritation by making loud noises (e.g., in connection with enforced quarantine measures); …*
	1.4.3 Having a say in the way health-related requirements are implemented	This sub-category gathers evidence on what opportunities people with PIMD have regarding their involvement in the implementation of health-related requirements and how they make use of them. Overlaps with subcategories 2.1.4, 2.2.1 and 2.3.3 are expected.	Field study Delphi 1	*demanding one’s own preferences regarding brand of tooth paste; indicating that physiotherapeutic measures cause pain (desire for alternative application).…*
2. Health-Related Information in Support of People with PIMD				
2.1 Engaging with health-related information by proxy				
	2.1.1 Assessing health situation and needs by proxy	This subcategory gathers evidence on requirements for professional (i.e., pedagogical, possibly also nursing or therapeutic) action that come up in connection with assessing health situations and needs by proxy. Overlaps with subcategories 1.3, 1.4 and 2.3 are expected.	Online survey Delphi-1 Field study	*using observation tools or pain detection instruments; contextualising health situation in life course; results from online-survey (A1); …*
	2.1.2 Searching for and assessing health-related information by proxy	This subcategory gathers evidence on requirements for professional (i.e., pedagogical, possibly also nursing or therapeutic) action that come up in connection with searching for and assessing the relevance of information by proxy. Overlaps with subcategories 1.3 and 2.3 are expected.	Online survey Delphi-1 Field study	*searching for information on specific vulnerabilities of p/w PIMD; results from online-survey (A1, B4 & C1); …*
	2.1.3 Passing on health-related information by proxy	This subcategory gathers evidence on requirements for professional (i.e., pedagogical, possibly also nursing or therapeutic) action that come up in connection with passing on information by proxy. Overlaps with subcategory 1.4 are expected.	Field study Online survey Delphi-1	*passing on relevant health-related information to health staff or family members; …*
	2.1.4 Raising awareness on health-related issues	This subcategory gathers evidence on requirements for professional (i.e., pedagogical, possibly also nursing or therapeutic) action that come up in connection with raising awareness on certain health-related issues. Overlaps with subcategory 1.4 are expected.	Field study Online survey Delphi-1	*Raising awareness on necessity of maintaining health lifestyle; results from online-survey (A1); …*
2.2 Illustrating health-related information & facilitating understanding				
	2.2.1 Considering individual communication modalities and needs	This subcategory comprises evidence on the requirement to consider different and individual communication needs and modalities of people with PIMD when communicating health-related information. Overlaps with subcategory 2.3 are expected.	Online survey Field study Delphi-1	*results from online-survey (A4, B1 & C2); using pictograms to indicate obligation to wear a mask; …*
	2.2.2 Considering individual sensory modalities	This subcategory comprises evidence on the requirement to give an opportunity to actively engage with the information. Overlaps with subcategory 2.3 are expected.	Field study Online survey Delphi-1	*using smells to indicate the requirement to apply disinfection; reminding p w/PIMD to do exercise by moving her feet; …*
	2.2.3 Giving the opportunity to actively engage with the information	This subcategory comprises evidence on the requirement to give an opportunity to actively engage with the information. Overlaps with subcategory 2.3 are expected.	Field study Online survey	*putting the toothbrush in the person’s hand;* *l* *etting the person wash his/her own hands; haptic exploration; …*
2.3 Joint engagement with health-related information				
	2.3.1 Exploring unfamiliar health-related situations together	This subcategory comprises evidence on how people with PIMD and their supporters explore unfamiliar health-related situations together (including the requirements that arise from them).	Field study Delphi-1	*learning how to deal with uncertainty in the beginning of the pandemic; mutual psycho-social support; …*
	2.3.2 Intersubjective/intercorporeal communication	This subcategory comprises evidence on the significance of intersubjective/intercorporeal communication. Overlaps with subcategories 2.1 and 2.2 are expected.	Field study Online survey	*unconscious perception of the needs of the other person; results from online-survey (B3 & B4); …*
	2.3.3 Finding a common “rhythm” in implementing health-related requirements	This subcategory comprises evidence on how people with PIMD and their supporters find compromises and agreements on the way health-related requirements are carried out (e.g., by finding compromises). Overlaps with subcategory 1.4.3 are expected.	Field study	*making physio-therapeutic interventions more pleasant by listening to music; Corona-testing may involve tearing apart the plastic packaging; …*
